# Reproductive tract infections in women seeking abortion in Vietnam

**DOI:** 10.1186/1472-6874-9-1

**Published:** 2009-01-29

**Authors:** Nguyễn Mỹ Hương, Jørgen Kurtzhals, Đỗ Thị Thu Thủy, Vibeke Rasch

**Affiliations:** 1 Institute of Population and Development Studies, General Office of Population and Family Planning, Ministry of Health, Vietnam; 2 Center for Medical Parasitology, Department of Clinical Microbiology, Rigshospitalet, Copenhagen University Hospital, Copenhagen, Denmark; 3 Hải Phòng Phụ-Sản Hospital, Hải Phòng, Vietnam; 4 Department of International Health, Immunology and Microbiology, University of Copenhagen, Copenhagen, Denmark

## Abstract

**Background:**

Women requesting abortion are at increased risk of developing RTI complications. However, RTI control in many resource-poor countries including Vietnam have been faced with logistical and methodological problems due to lack of standardized definitions of RTIs, lack of well-validated diagnostic criteria, lack of accurate laboratory tests, and lack of diagnostic equipment and skills. This article investigates the prevalence of RTIs among Vietnamese abortion-seeking women, to evaluate the available diagnostic techniques, and to assess antibiotic resistance among aetiological agents of RTI.

**Method:**

The study was conducted in Phu-San hospital (PSH) from December 2003 through April 2004 among 748 abortion clients. A structured questionnaire was used to collect data on socio-economic and reproductive characteristics. Specimens were collected for laboratory analyses of chlamydia, gonorrhoea, trichomoniasis, vaginal candidiasis (VC), bacterial vaginosis (BV) and syphilis. To assess the validity of the obtained results, the study was repeated among 100 women and the duplicate samples were analysed at PSH and Copenhagen University Hospital (CUH).

**Results:**

In all 54% of the women were diagnosed as having an RTI, including 3.3% with sexually transmitted infections. Endogenous infections were most prevalent (VC 34% and BV 12%) followed by chlamydia (1.3%) and trichomoniasis (0.7%). The sensitivity of culture for VC and BV was 30% and 88%, respectively, when tests in PSH were measured against tests in CUH. Antibiotic resistance was common among bacterial isolates.

**Conclusion:**

RTIs are common among women seeking abortion. The presence of RTIs is associated with an increased risk of developing iatrogenic infections, routine administration of prophylactic antibiotic to all women undergoing abortion should be considered. However, the choice of routine prophylactic antibiotics should be based on relevant surveillance data of antibiotic resistance. Moreover, since the accuracy of diagnosis is doubtful and to address the problem of under-diagnosed and treated RTIs new investment in diagnostic facilities with simple performed microscopy or improved rapid tests should also be taken into consideration.

## Background

With an annual incidence of 340 million STI cases globally and many more endogenous and iatrogenic infections, reproductive tact infections (RTIs) are considered a global public health issue [1]. In resource-poor countries, where 75%–85% of these new cases occur, RTIs are among the five most common health problems leading to contact with the health system (1). RTIs entail a heavy toll on women, if untreated they can lead to pelvic inflammatory disease (PID), which can cause long-term sequelae, such as tubal infertility and ectopic pregnancy [2,3]. RTI control in many resource-poor countries have been faced with logistical and methodological problems due to lack of standardized definitions of RTIs, lack of well-validated diagnostic criteria, lack of accurate laboratory tests, and lack of diagnostic equipment and skills [1]. Therefore, the burden of RTIs may be substantially underestimated [4] and is rarely on the list of public health priorities [5].

Women requesting abortion are at increased risk of developing RTI complications. Hence, in high income countries, where proper procedures to prevent and control hospital acquired infections are implemented and adhered to, induced abortion is associated with a 5–10% risk of post-abortion infection, including PID [6-8]. The risk of infection and PID is further increased in the presence of chlamydia, gonorrhoea and bacterial vaginosis (BV) at the time of induced abortion as e.g. reported in a Swedish study where BV was found to be a risk factor for post-abortion uppergenital-tract infection [9,10]. The risk of developing iatrogenic infections in relation to the abortion procedure may be assumed to be higher in resource-poor settings where hospitals may lack appropriate antimicrobial therapy and infection control practices.

RTIs are common in Vietnam. According to national data, number of gynaecological examination in 2006 was 11,464,896; of which 5,000,927 cases were treated [11]. Community-based studies have reported RTI prevalence rates ranging from 50% to 80% [12,13]. Induced abortion is also a prevailing problem. According to official reports, more than half a million induced abortions were performed in Vietnam in 2003, and the abortion rate was 26 per 1,000 women [14]. However, it is believed that the number of abortions are seriously underreported since the reports do not cover a number of unregistered abortions as well as abortions performed in private health facilities [15,16]. Though vacuum aspiration that is used for first trimester pregnancies is considered relatively safe with little trauma to the cervix and uterus, unsafe abortion still accounts for about 11.5% of maternal deaths in Vietnam [17]. The prevalence of RTI which may hamper the safety of the procedure is unknown due to poor testing facilities, a weak technical ability of personnel, inadequate training, and lack of supervision and quality control [18]. Studies have indicated that syndromic diagnosis of RTIs may be a problem among abortion clients [19] because RTI detection and prevention only receive little attention from both the health staff and their clients in abortion settings [20]. Thus, the present study aimed to determine the prevalence of RTIs among Vietnamese abortion-seeking women, to evaluate the available diagnostic techniques, and to assess antibiotic resistance among aetiological agents of RTI.

## Methods

### Study subjects

The study was carried out in two time periods: the prevalence study from December 2003 through April 2004 among 748 abortion clients and the validation study in two separated weeks in January and April 2005 among 100 women. Women attending Phu-San Hospital in Haiphong for induced abortions between December 2003 and April 2004 were invited to participate in the study. During the study period, of 811 women seeking pregnancy termination at the hospital, 748 women had an induced abortion performed and were thus eligible to participate in the study. They were invited and all of them agreed to take part in the study. In the second period, two set of samples were collected among 100 abortion clients during one week in January and one week in April 2005.

### Data collection

A structured questionnaire was used to collect data about the women's socio-economic and reproductive characteristics.

### Sample collection, storage, and transport

High-vaginal and endocervical specimens for RTI testing were collected during vaginal examinations using standard procedures. A blood sample (2–3 ml) was taken for serological tests. Vaginal fluid from the posterior vaginal fornix was tested for pH and prepared for a whiff test at the abortion clinic. Wet mount preparations and Gram stained smears for microscopic examination for *Trichomonas*, lactobacilli, polymorphonuclear leucocytes and vaginal epithelial cells were performed and evaluated at the PSH Department of Microbiology using national standard laboratory guidelines. Swabs of vaginal fluid were cultured as described below. After the cervix was wiped free of vaginal discharge, specimens for detection of *Chlamydia trachomatis *were taken with a swab and placed in transport medium. To determine the effectiveness of on-site testing, neither clinicians nor lab-technicians received any additional training in making on-site detection of vaginal discharge. They were instead asked to perform and interpret the observed result as they used to by using syndromic management of STIs and laboratory guidelines from the hospital and from the department of microbiology. As a form of quality control, the head of laboratory service examined all positive results personally.

To verify the results, the study was repeated among another 100 abortion clients, from whom duplicate specimens were collected during the two one-week periods (validation study). One of the two sets of samples was analysed in PSH using the same techniques applied in the prevalence study. The other set of specimens was stored at 4°C for up to one week and sent by air on ice (transit time 24 hours) to the Department of Clinical Microbiology, Copenhagen University Hospital (CUH) immediately after the last specimen had been collected. Samples for ordinary culture and sensitivity testing were kept in Stuart's transport medium (SSI, Copenhagen, Denmark) during transport. Before shipment, direct smears were Gram stained, and swabs for *Neisseria gonorrhoea *isolation were incubated in InTray™GC (BIOMED Diagnostics, San Jose, CA, USA) immediately after sample collection. These samples were pre-incubated at 37°C before shipment. Endocervical specimens for detection of *C. trachomatis *were shipped in transport medium included in the Amplicor kit (see below). The laboratory results from CUH were considered as the reference (gold-standard).

### Prevalence of RTI

The criteria used for laboratory diagnosis of RTI are presented in Table 1.

**Table 1:** Laboratory test performed and criteria used for diagnosis of RTI

**Organism/syndrome**	**Test performed**	**Diagnostic criteria**
Bacterial vaginosis	KOH whiff test	Positive whiff test
	Vaginal pH	Vaginal pH > 4.5
	Vaginal smear for wet mount preparation and Gram staining	Clue cells > 20% of all cells observed on wet preparation or Gram stain
	Vaginal microbiologic culture	Positive culture (cervical or vaginal) for relevant microorganisms *
*Chlamydia trachomatis*	SD Bioline Chlamydia dipstick test	Positive test
	Chlamydia PCR (validation study)	Positive test
Syphilis	Abbott Determine Rapid Syphilis TP assay	Positive test
Trichomonas	NaCl wet mount preparation	Trichomonas observed on wet mount preparation
	InPouch™ TV culture (validation study)	Positive culture
Candidiasis	NaCl wet mount preparation Vaginal gram stain	Fungal elements observed on wet mount preparation; and/or 3–5 budding yeasts observed per oil immersion field on Gram stain.
	Vaginal microbiologic culture	Culture of *C. albicans*
Gonorrhoea	Endocervical smear for Gram staining	Gram negative intracellular diplococci
	Endocervical microbiologic culture InTray™ GC (validation study)	Positive culture

* Including Gardnerella, *Pseudomonas spp*., and enterobacteriaceae.

All tests were done in PSH Department of Microbiology using routine methods unless otherwise indicated.

#### The whiff test

A swab of the vaginal fluid was dipped in 1 ml of 10% KOH and mixed for 3–5 minutes. The test was interpreted as being positive, i.e. suggestive of *G. vaginalis*, if a fishy odour was noted.

#### Wet mount preparation and examination

A vaginal swab was mixed with 1 ml of saline and one drop was placed on a slide for examination under bright-field microscopy at × 200–400 magnification. It was examined for the presence of motile trichomonads, fungal hyphae, granulocytes and clue cells.

#### Gram staining

Gram stained smears were prepared from vaginal swabs and examined under oil immersion at × 1000 magnification. If there were > 3 budding yeasts per oil immersion field, the woman was diagnosed as having candidiasis; the presence of ≥ 20% clue cells per oil immersion field indicated BV. Gonorrhoea was identified with the presence of Gram negative intracellular diplococci.

### Culture

All culture media employed in PSH were prepared locally using agar plus nutrient broths. The specimens were spread on 5% rabbit blood agar, chocolate agar, and Sabouraud and incubated at 37°C for 24–72 hours. Blood agar and Sabouraud media were incubated in air and chocolate agar plates in a humidified atmosphere with 10% carbon dioxide. The following microorganisms were isolated and identified: *N. gonorrhoea*, *Candida albicans*, *G. vaginalis*, and pathogenic Gram negative rods. For the validation study, the samples were also tested for *T. vaginalis *by using a selective culture system InPouch™ TV (Biomed) according to manufacturer's instruction.

The samples analysed at CUH were plated on horse blood agar, chocolate agar, and human blood agar (HBT, all from SSI) and incubated in a humidified atmosphere with 5% CO_2 _for 48 hours. Colonies typical of yeast, faecal flora, *Streptococcus agalactiae*, and *G. vaginalis *were identified using standard procedures. Antibiograms were prepared on Danish Blood Agar (SSI) using Neo-sensitabs (Rosco, Taastrup, Denmark). The InTray™GC was inspected for gonococcal colonies. Gram stained smears were screened for clue cells and granulocytes.

#### Detection of C. trachomatis

*C. trachomatis *was identified by Chlamydia dipstick quick test (SD BIOLINE Chlamydia, Standard diagnostic, INC, Yongin-Si, Gyeonggi_Do, South Korea). The test is an immunochromatographic antigen detection assay, which utilizes monoclonal antibodies specific to *C. trachomatis *antigen. The cervical swabs were processed and tested according to the directions provided by the manufacturer. Results were read after 15 minutes.

In CUH, *C. trachomatis *infection was detected by PCR using the Cobas Amplicor system (Roche Diagnostics Systems, Inc., Branchburg, NJ, USA).

#### Serological test for syphilis

Serum samples were centrifuged at 12,000 rpm for 5 minutes and 50 μl of the supernatant was used for syphilis testing using Abbott Determine Rapid Syphilis TP assay (Abbott Laboratories, Abbott Park, USA) in accordance with the manufacturer's instructions.

### Additional investigations

Because of concerns about transport of the samples of gonococcal culture, eight freeze-dried isolates of *N. gonorrhoea *were sent from CUH to PSH where they were incubated and identified using standard methods. As quality control, the same isolates were kept in CUH under conditions similar to that in air-transport and cultured at the same time as in PSH

### Statistical analyses

Data were entered twice by two different operators using the software program Epi Data 3.0 (Danish Society of Public Health, Copenhagen, Denmark). The two data sets were compared and the questionable entries reconciled. Statistical analysis was performed by the Statistical Package for the Social Sciences (SPSS 11.5, SPSS Inc.). Data were analysed through descriptive tables of the variables studied. The validation of data from both study periods was evaluated through basic frequency measures. Binomial confidence intervals were calculated using Bayesian statistics with no prior assumptions. The validity of the tests performed in PSH was then assessed by comparing the sensitivity, specificity, positive and negative predictive values of each PSH lab-test vs. the corresponding CUH lab-test.

### Ethical considerations

The study was approved by The Danish National Committee on Biomedical Research Ethics. All women and health providers were informed about the study objectives, and enrolment required signed, informed consent. The confidentiality and anonymity of all participants were ensured. All participants were informed about their gynaecological status and if a woman was found to have treatable RTI, the doctor provided appropriate therapy using standard treatment guidelines based on available antibiotics.

## Results

### Patient characteristics

The mean age of 748 study participants was 29 years (SD 7) with the most prevalent age group being 20–29 years (56%), followed by 30–39 years. The mean number of previous pregnancies was 3 (range: 0 to 10). The mean number of previous abortions per woman was 2 (range: 0 to 9). Most of the clients, 690 (92%), were less than 12 weeks pregnant with a mean gestational age of 7 weeks (range of 1–25). Of the total, 71% were married/cohabiting, of whom the mean number of children was 1.56 (SD 0.6). About one third of women had never had children. The majority of the women had given birth once or more (68%). A large majority (75%) were living in urban areas. Similar characteristics were found among women enrolled in the validation study (Table 2).

**Table 2:** Selected socio-demographic characteristics of the respondents

**Socio-demographic variables**	**Prevalence study** **N = 748**	**Validation study** **N = 100**
**Age (year)**		
< 20	2.6%	5%
20–29	55.8%	45%
30–39	31.2%	33%
≥ 40	10.4%	17%
***Mean (SD) ***	***29.3 (± 7) years***	***30.6 (± 7.7) years***
**Marital status**		
Married/cohabiting	71.5%	79%
Single	28.5%	21%
**Pregnancy (including present pregnancy)**		
1	24%	20.5%
2	22.5%	26.5%
≥ 3	53.5%	53%
***Mean number of pregnancy (SD)***	***3.1 (± 1.8)***	***3.1 (± 1.8)***
**Number of living children**		
**0**	31.4%	31.0%
**1**	33.4%	32.6%
**2**	31.7%	32.1%
**>= 3**	3.4%	4.3%
***Mean number of living children ***	***1.56 (± 0.6) children ***	***1.52 (± 0.6) children***
**Abortions (including present abortion)**		
1	47.9%	45.9%
2	28.6%	21.3%
3	15.1%	17.6%
≥ 4	8.4%	15.2%
**Residence**		
Urban	74.9%	79%
Rural	25.1%	21%

The prevalence of RTIs was 54.4%, of which endogenous RTIs accounted for 51.2% and STIs accounted for the remaining 24 cases (3.3%) (Table 3 and Table 4). The principal infectious microorganism was *C. albicans*, which was identified in 255 women (34%). Less frequent was BV (12%) including 8 cases (1.1%) with enterobacteriaceae (*Eschericia coli *and *Proteus mirabilis*). The most prevalent co-infection was candidiasis with BV that was found in 38 patients (5%). The most prevalent STI was *C. trachomatis *(2.5%), followed by trichomoniasis (0.7%). Syphilis was found in one woman (0.1%), and eight women were found to have both *C. trachomatis *and endogenous RTI (1.1%). No *N. gonorrhoea *was detected. There was no difference in the rates of STIs and endogenous infections when stratify for age parity and marital status.

**Table 3:** The prevalence of RTI among abortion clients

**Reproductive tract infections**	**RTI prevalence N = 748**		**Validation study N = 51**			
	
	**N (%)**	**95% CI** **%**	**PSH** **N (%)**	**95% CI** **%**	**CUH** **N (%)**	**95% CI** **%**
**Any RTI**	**407 (54.4)**	50.8–58.9	**29 (57)**	42–71	**35 (69)**	54–81
**Endogenous RTI**	**383 (51.2)**	47.6–54.8	**29 (57)**	42–71	**34 (67)**	52–79
VC	255 (34.1)	30.7–37.6	22 (43)	29–58	17 (33)	21–48
BV^2^	90 (12.0)	9.8 – 14.6	7 (14)	6–26	10 (20)	10–33
Co-infection^3^	38 (5.1)	3.6 – 6.9	0	0–7	7 (14)	6–26

1. Endogenous RTI (validation study): based on bacterial and fungal culture plus microscopy of Gram-stained smears (N = 51)

2. Bacterial vaginosis (BV) (prevalence study): including 8 cases (1.1%) of Enterobacteriaceae (*E. coli *and *P. mirabilis*)

3. Co-infections: both vaginal candidiasis (VC) and BV

**Table 4:** The prevalence of STI among abortion clients

**Organisms**	**Prevalence study**		**Validation study N = 100 (%)**			
	
	**N = 748 (%)**	**95%CI**	**PSH**	**95%CI**	**CUH**	**95%CI**
**STI**	**25 (3.3)**	2.2–4.9	**2 (2)**	0–7	**6 (6)**	2–13
Trichomoniasis	5 (0.7)	0.2–1.6	0 (0)	0–4	0 (0)	0–4
*Chlamydia *infection	19 (2.5)	1.5–3.9	2^1 ^(2)	0–7	6^2 ^(6)	3–13
Gonorrhea	0 (0.0)	0.0–0.5	0 (0)	0–4	0 (0)	0–4
Syphilis	1 (0.1)	0.0–0.7	0 (0)	0–4	Nd^3^	

1. Both patients had candida.

2. Two also BV and one also candida

3. Nd, Not done

Due to problems of transport, only 51 samples were accepted for routine culture and sensitivity testing in CUH, whereas microscopy of Gram-stained smears plus analyses for *Chlamydia *and gonococci could be done for all 100 samples. The prevalence of RTI detected by laboratory investigations in PSH and CUH was of the same magnitude as that in the prevalence study. Of the 51 cultured samples, 29 in PSH and 35 in CUH were suggestive of at least one RTI corresponding to an overall RTI prevalence of 57% and 69%, respectively.

The prevalence of vaginal candidiasis detected by culture was 43% in PSH and 33% in CUH. The prevalence of BV was 14% in PHS and 20% in CUH. In some cases, coagulase-negative staphylococci were found in Hai phong whereas enterococci or streptococci were found in Copenhagen in the parallel samples. In 7 cases, lab technicians in PSH only reported *C. albicans*, while both *C. albicans *and Gram negative rods were found in the parallel samples in CUH.

Using the rapid antigen detection assay, 2 women were diagnosed with chlamydial infection compared with 6 infections detected by PCR. The 2 cases detected by antigen detection in PSH were different from the 6 cases detected by PCR. This test result also indicated that *Chlamydia *infection occurred more often in older age-groups than expected. None of the 8 freeze-dried N.gonorrhoea cultures could be cultured in PHS suggesting a serious lack of sensitivity for gonococcal infections. Seven of 8 freeze dried samples stored in similar cryoboxes for a time corresponding to air transport from CUH to PSH could be cultured in CUH.

Laboratory test results in CUH were used as a gold-standard to assess the validity of diagnoses obtained at PSH (Table 5). The culture used in PSH had a relatively high sensitivity for VC (88%) whereas the specificity was 79%. When focusing on BV, the sensitivity and specificity of the test used at PSH were 30% and 90%, respectively in comparison with the tests used at CUH. Compared with the PCR assay, the sensitivity and the specificity of the *Chlamydia *rapid test used at PHS were 0% and 98%, respectively.

**Table 5:** Accuracy of lab-test results of PSH compared with that of CUH of selected RTIs (validation study)

**PSH**	**CUH**		**Total (N)**	**Sensitivity**	**Specificity**	**PPV**	**NPV**
						
	Positive	Negative					
**Vaginal candidiasis**			51	88	79	68	93
Positive	15	7					
Negative	2	27					
**Bacterial vaginosis**			51	30	90	43	84
Positive	3	4					
Negative	7	37					
**Chlamydia**			100	0	98	0	94
Positive	0	2					
Negative	6	92					

Note: only tests with complete data were included in this analysis.

PPV = positive predictive value

NPV = negative predictive value

Antibiotic resistance were assessed in the samples analysed at CUH (table 6). The following species were isolated from the vaginal swabs: G. vaginalis, E. coli, K. pneumoniae, Enterobacter spp and Enterococcus faecalis. Gram negative bacteria (E. coli, K. pneumoniae, Enterobacter) were found to be resistant to ampicillin and sulfamethoxazole with trimetroprim whereas most of the species isolated were sensitive to cefuroxime, ciprofloxacin and gentamicin. Enterococci were not considered pathogenic but were included here to demonstrate good sensitivity to ampicillin and vancomycin.

**Table 6:** Bacteria isolated from vaginal swabs (CUH)

Number of isolates	Species	**Sensitivity^1 ^(number of sensitive isolates)**						
		
		Pen	Ampi.	Cefur.	S+T	Cipro	Genta	Vanco
2	*G. vaginalis*	-	-	-	-	-	-	-
5	*E. coli*	-	0	5	0	5	5	-
3	*K. pneumoniae*	-	0	3	1	3	3	-
2	*Enterobacter spp.*	-	0	0	1	2	1	-
10	*Enterococcus faecalis*	-	10	-	-	-	-	10

^1^Pen, penicillin G; Ampi, ampicillin; Cefur, cefuroxime; S+T, sulphametoxazole with trimethoprim; Cipro, ciprofloxacin; Genta, gentamicin; Vanco, vancomicin.

## Discussion

The present study documents that RTIs is common among Vietnamese women requesting abortion. The findings from the present study reveal that abortion-seeking women apparently have a relatively high prevalence of RTI compared to the general population of women of reproductive age. Other Vietnamese studies have shown a similar high prevalence of RTIs [19,21,22]. Our findings are in line with a cohort study from where has documented that presence of bacterial vaginosis and *M. hominis *were twice as common in women who had had an induced abortion than among the control group [23]. Further, among the women who have undergone an abortion, a history of PID is four-fold that of women who have not undergone an abortion [23]. The endogenous infections accounted for the vast majority of the identified RTIs: VC was identified in 39–44% of the samples analyzed at PSH and in 47% of the samples analyzed in CUH; BV was found in 14–17% of the samples analyzed at PSH and in 33% of the samples analyzed at CUH. This high prevalence may reflect a change in the nature of vaginal discharge caused by pregnancy, douching practice, use of antibiotic prior to specimen collection, or delay in culturing the vaginal specimens. The observed prevalence rates are considerably higher than what has been found in other Vietnamese studies [19,22,24]. A possible explanation for the high endogenous infection prevalence found in our study may be a more pronounced tendency towards using vaginal douching in the setting studied [25]. Such an association between vaginal douching and endogenous RTIs has previously been demonstrated [22,26,27].

According to the validation study the STI prevalence was low, only 2% of the women were diagnosed as having an STI at PSH whereas the same applied for 6% when the analyses were performed at CUH. Although the test results for Chlamydia and gonorrhoea in PSH may have suffered from a lack of sensitivity, the STI prevalence rate found in the current study is in accordance with the findings from the aforementioned studies, which reported STI rates varying from 1% to 6% and which also found very low rates (0–1%) of syphilis and gonorrhoea [21,22]. This relatively low STI prevalence may reflect that sexual risk taking is uncommon in Vietnam as suggested from a study from Northern Vietnam [28]. Similarly, a study conducted among adolescents in Ho Chi Minh City revealed that a serious, loving relationship with definite commitment to marriage is valued by young people [29]. Our study population comprised relatively young women, 58% were under 30 years old. However, the poor performance of chlamydia antigen testing and gonococcal culture suggests that better validated studies are indicated in order to confirm the low incidence of STIs.

Antibiotic resistance, especially against ampicillin and sulphametoxazol with trimethoprim, was common in the present study. These two drugs are commonly used for treating urogenital infections in Vietnam. The resistance pattern resembles that reported in two other Vietnamese studies focusing on community-acquired pneumonia and agricultural animals [30,31]. One explanation for the observed resistance towards ampicillin and sulphametoxazol with trimethoprim is that these two types of antibiotics, in contrast to cephalosporins, quinolones and aminoglycocides, which had all retained their activity, are often bought over the counter without any prescription [30]. Another likely contributing factor could be that patients as well as health care providers rely heavily on antibiotics, which are often being used without the establishment of a proper clinical diagnosis [25]. Our findings suggest that the first line treatment of infections with Gram negative bacteria needs to be changed. However, the data are limited and more studies are needed to guide a change in first-line antibiotic treatment.

In relation to abortion, it is well known that the presence of gonorrhoea and chlamydia in the cervix prior to instrumentation of the uterus increases the risk of postabortion infection and associated long-term complications such as ectopic pregnancy and infertility. Moreover, it has also been documented that the presence of BV increases the incidence of PID after first trimester abortions [9]. Diagnosis and treatment is thus critical to the safety and quality of abortion services. In that relation it should be acknowledged that treatment with metronidazole in conjunction with first-trimester abortion has proven to reduce the postoperative infection rate more than three times among women with BV [10]. Hence, to address the risk of PID and associated complications among women requesting abortion, it should be considered to routinely administer prophylactic antibiotics to all women undergoing abortion. However, it is of paramount importance that antibiotic resistance is assessed and addressed; otherwise prophylactic antibiotics will be useless.

The major limitations of the present study is the possibility of misclassification i.e. a woman with RTI classified as not having infection and vice versa. To minimize such misclassification and to enhance the accuracy and precision of each test, the head of laboratory service examined all positive results personally. Furthermore, the diagnostic abilities of the laboratory staff in Vietnam were scrutinised against a "gold standard" where identical samples from 100 women were analysed at PHS and at CUH. It may, however, be argued that no laboratory test can be considered the "gold standard" and it may be questioned whether it is right to use the test from CUH as a golden standard since these analyses were performed with a relatively small sample (100 cases). While confirming the high prevalence of RTIs, the validation study showed a poor agreement between the specific RTIs identified in CUH and PSH. Especially, the sensitivity of the culture for *C. albicans *conducted in PSH was low which, may have led to inappropriate treatment of 70% of the infected women. Contrary, the culture result for BV had relatively high sensitivity but low specificity. Erroneous diagnosis of BV as well as other RTIs is a great concern and seems to be prevailing regardless of whether recommended technologies are used or not. The study was further a clinical-based study conducted to assess the prevalence of RTIs among women seeking termination of unwanted pregnancy. The study may thus, be critisized for its potential selection bias. Hence, the internal representatively is likely to be high whereas the external representatively can be questioned since women who are seeking abortion care at one hospital in Northern Vietnam may differ from women who are seeking abortion care in another part of Vietnam. Thus, there is no assurance that the findings from this study will be identical with studies from other abortion settings. To increase the internal validity, all interviewers received comprehensive training to encourage rural or young unmarried women to participate in the study

At present the syndromic approach is considered the most feasible approach at health centers in resource-poor settings in the management of common RTIs and STIs. Even so, findings of various studies conducted to evaluate the performance of this approach have shown poor specificity and low positive predictive value for detection of BV as well as other RTIs [32,33]. Laboratory investigations play an important role in diagnosing RTIs – also in context of the syndromic approach. However, the findings from the present study indicate that laboratory based diagnoses of RTIs remain a challenge. A parallel study in the same population of women has found that factors hampering the use of the etiological approach include weak technical abilities, inadequate staff training, and a lack of supervision [20]. To overcome the difficulties in establishing reliable RTI diagnoses there is a need for the development of diagnostic facilities with simple performed microscopy or improved rapid tests or both.

## Conclusion

Since the accuracy of diagnosis is doubtful, to address the problem of under-diagnosed and treated RTIs new investment in diagnostic facilities with simple performed microscopy or improved rapid tests should also be taken into consideration. Additionally, presence of RTI is associated with an increased risk of developing iatrogenic infections; therefore, routine administration of prophylactic antibiotic to women undergoing abortion presently should be considered. Moreover, in the context of antibiotic resistance are common in Vietnam, the choice of routine prophylactic antibiotics should be based on relevant surveillance data of antibiotic resistance.

## Abbreviations

RTI: reproductive tract infection; STI: sexually transmitted infection; PSH: Phu-San hospital; PID: pelvic inflammatory disease; BV: bacterial vaginosis; CUH: Copenhagen University Hospital; SD: standard deviation; CV: vaginal candidiasis; PCR: polymerase chain reaction; CI: confidence interval; PPV and NPV: positive and negative predictive value.

## Competing interests

The authors declare that they have no competing interests.

## Authors' contributions

NMH contributed to the study design, was responsible for data collection and data analysis and drafted the manuscript. VR supervised the study and helped to draft the manuscript. JK conducted the laboratory tests in CUH and helped drafting the manuscript. DTTT supervised the laboratory tests conducted in PSH and helped drafting the manuscript. All authors read and approved the final manuscript.

## Pre-publication history

The pre-publication history for this paper can be accessed here:


